# Supplementary material for: Locomotion modulates specific functional cell types in the mouse visual thalamus
Source: Nat Commun. 2018 Nov 19;9:4882. doi: 10.1038/s41467-018-06780-3 (PMC6242985; doi:10.1038/s41467-018-06780-3)
Supplement: Supplementary file 1 — Supplementary Information [file 41467_2018_6780_MOESM1_ESM.pdf]

File Name: Supplementary Information

Descriptions: Supplementary Figures

Title: Locomotion modulates specific functional cell types in the mouse visual thalamus

Authors: Çağatay Aydın [1,2], João Couto[1,2], Michele Giugliano[1,3,5,6,7], Karl Farrow[1,2,3], Vincent Bonin[1,2,3,4*]

[1]Neuro-Electronics Research Flanders, Kapeldreef 75, 3001 Leuven, Belgium.

[2]Department of Biology & Leuven Brain Institute, 3000 Leuven, Belgium.

[3]VIB, 3001 Leuven, Belgium.

[4]imec, 3001 Leuven, Belgium.

[5]Department of Biomedical Sciences, University of Antwerp, Antwerpen, Belgium.

[6]Brain Mind Institute, EPFL, Lausanne, Switzerland.

[7]Department of Computer Science, University of Sheffield, Sheffield, United Kingdom.

Correspondence should be addressed to V.B. (email: vincent.bonin@nerf.be).
These authors contributed equally: Çağatay Aydın, João Couto.

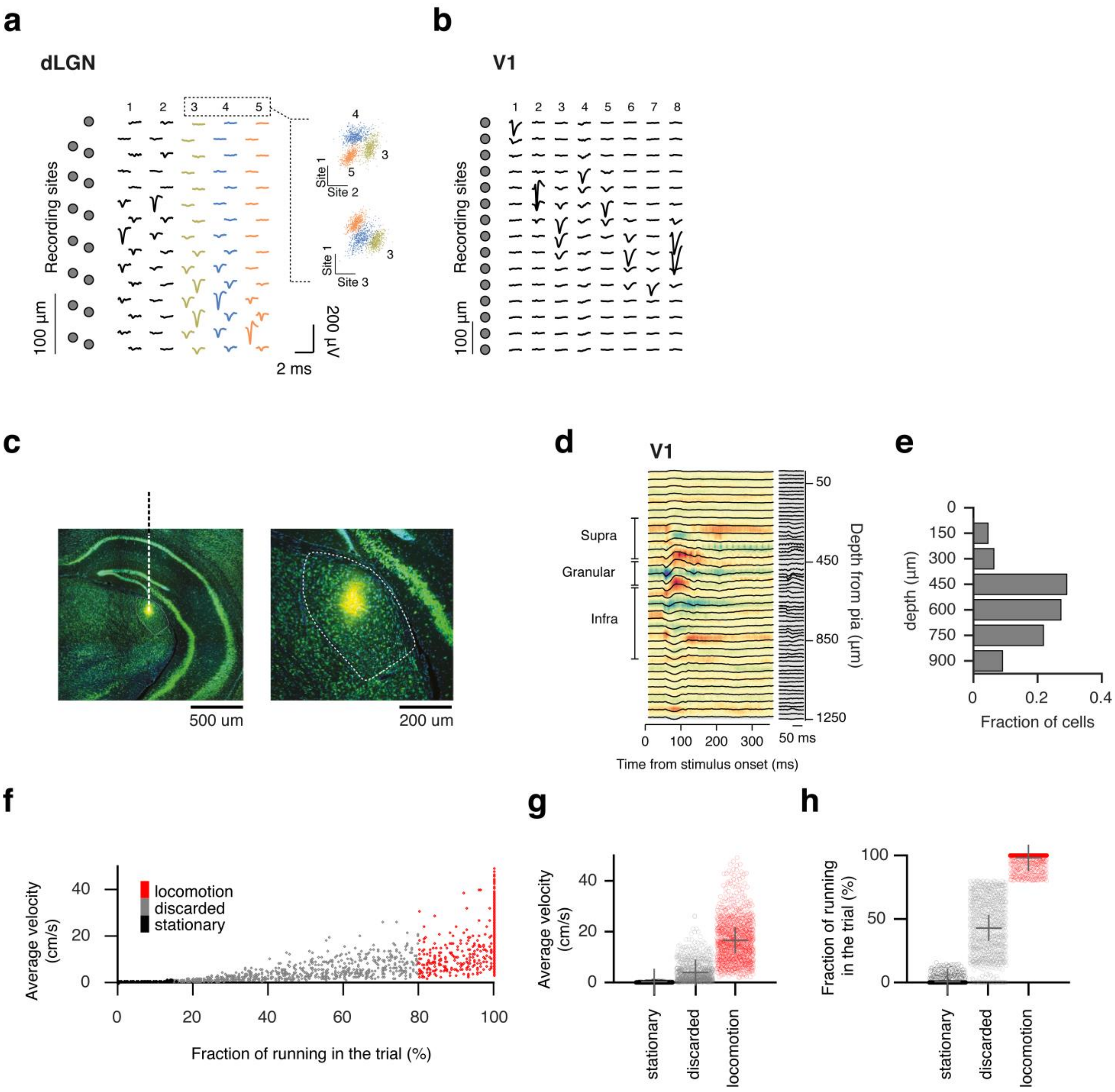

Supplementary Figure 1: (a) Illustration of the linear treadmill for the locomotion assay. Inlet represents the LCD screen in degrees. **(b)** Illustration of electrode site arrangement and example of simultaneously recorded single units dLGN (n=5 cells) and from V1 (n=8 cells), inlet represents the first principal component in 3 channels plotted against each other for 3 units in dLGN. **(c)** Example probe re-construction of the dLGN recording. Scale bar for the left panel is 200µm. Scale bar for the right panel is 500 µm. **(d)** Current source density analysis trigged by the onset of full screen contrast reversal stimulus to identify layer in V1 (recorded by neuropixel probe). **(e)** Distribution of the cells recorded from V1 across depth from pia. **(f)** Comparison of average animal velocity and fraction of running within trials for stationary (black), discarded (gray) and locomotion (red) trials. **(g)** Distribution of the average velocity for 3 trial types given in f. **(h)** Distribution of the fraction of running within the trial for the 3 trial types given in f.

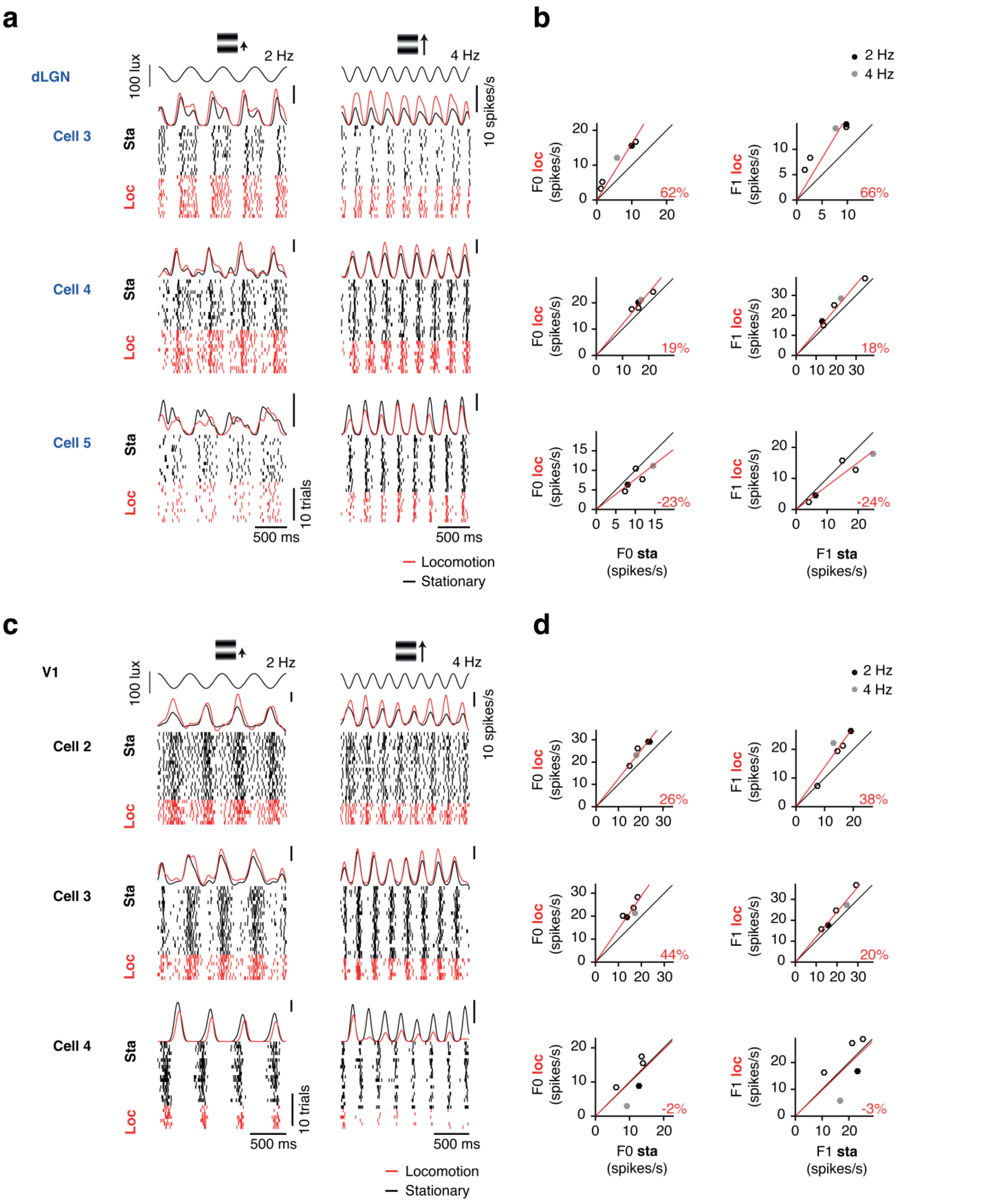

Supplementary Figure 2: (a) Spike rasters from 3 simultaneously recorded dLGN cells during the full field drifting gratings (temporal frequency: 2Hz; right, 4Hz; left). Locomotion (red) and stationary (black) trials are plotted independently. Scale bar for peristimulus time histogram is 10 spikes/s. Scale bar for spike rasters is 10 trials.**(b)** Mean firing rate (F0) and response (F1) amplitude in stationary and locomotion trials for 5 stimuli with different temporal frequencies for the cells in a. (62% increase, 19% increase and 23% decrease in firing rate: 66% increase, 18% increase and 24% decrease in response amplitude) **(c)** Spike rasters from 3 simultaneously recorded V1 cells. **(d)** Same quantification as b for V1 cells (28% increase, 44% increase and no change in firing rate: 38% increase, 20% increase and no change in response amplitude).

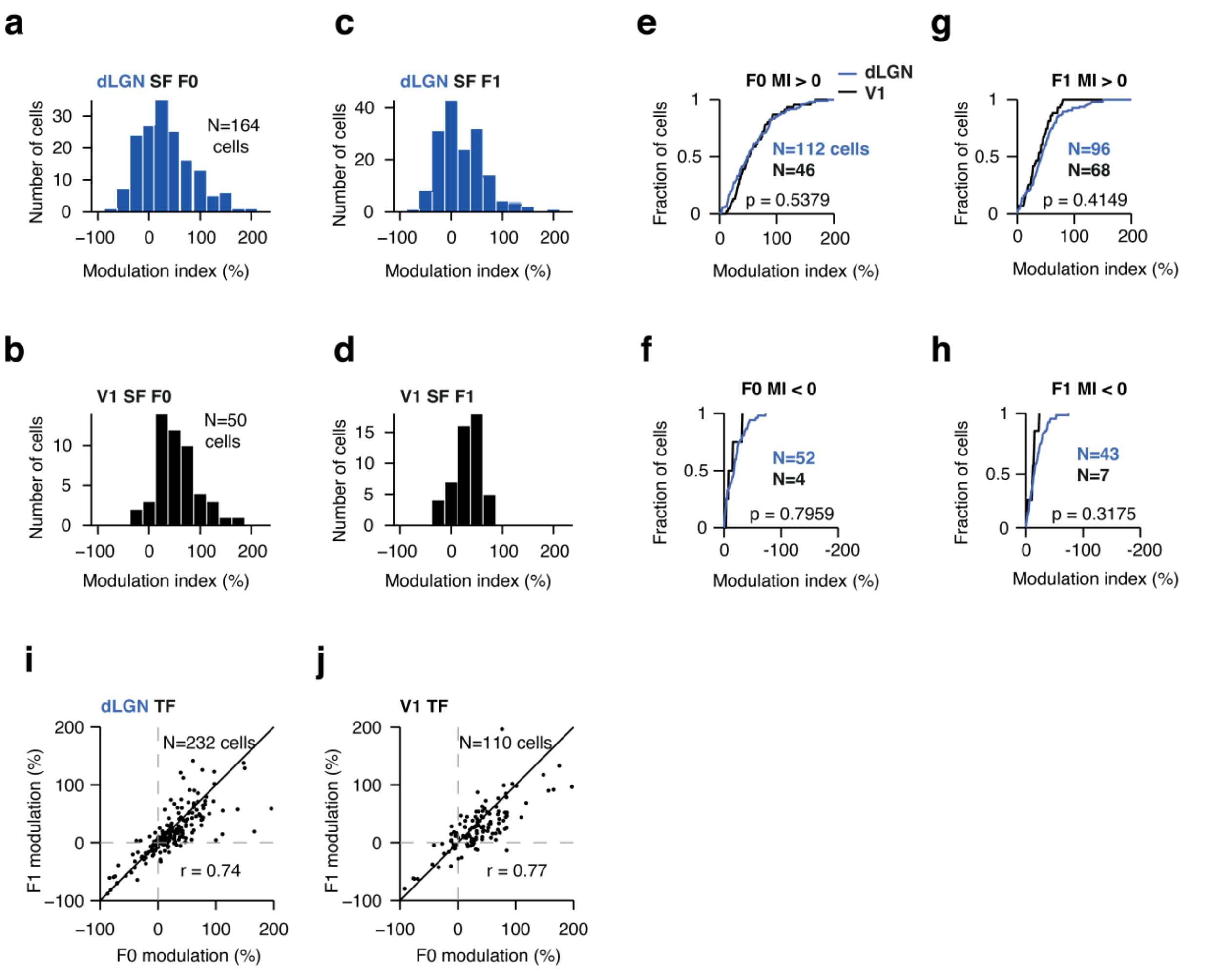

Supplementary Figure 3: (a) Distribution of firing rate (F0) modulations for dLGN cells (n=167 cells; in blue) for the spatial frequency experiments. **(b)** Same as panel a for V1 (n=44 cells; in black) **(c)** Distribution of response amplitude (F1) modulations for cells in a **(d)** Distribution of response amplitude (F1) modulations for cells in b. **(e)** Cumulative distribution of firing rate (F0) modulations for the cells with positive modulation index (MI>0) **(f)** Same as panel e for negatively modulated cells (MI<0). **(g)** Response (F1) modulations for the cells with positive modulation index (MI>0) **(h)** Same as panel g for negatively modulated cells (MI<0). **(i)** Comparison of modulation index values computed from firing rate and response amplitude for dLGN (N= 232 cells) to the temporal frequency stimulus **(j)** same as i for V1 cells (N=110 cells).

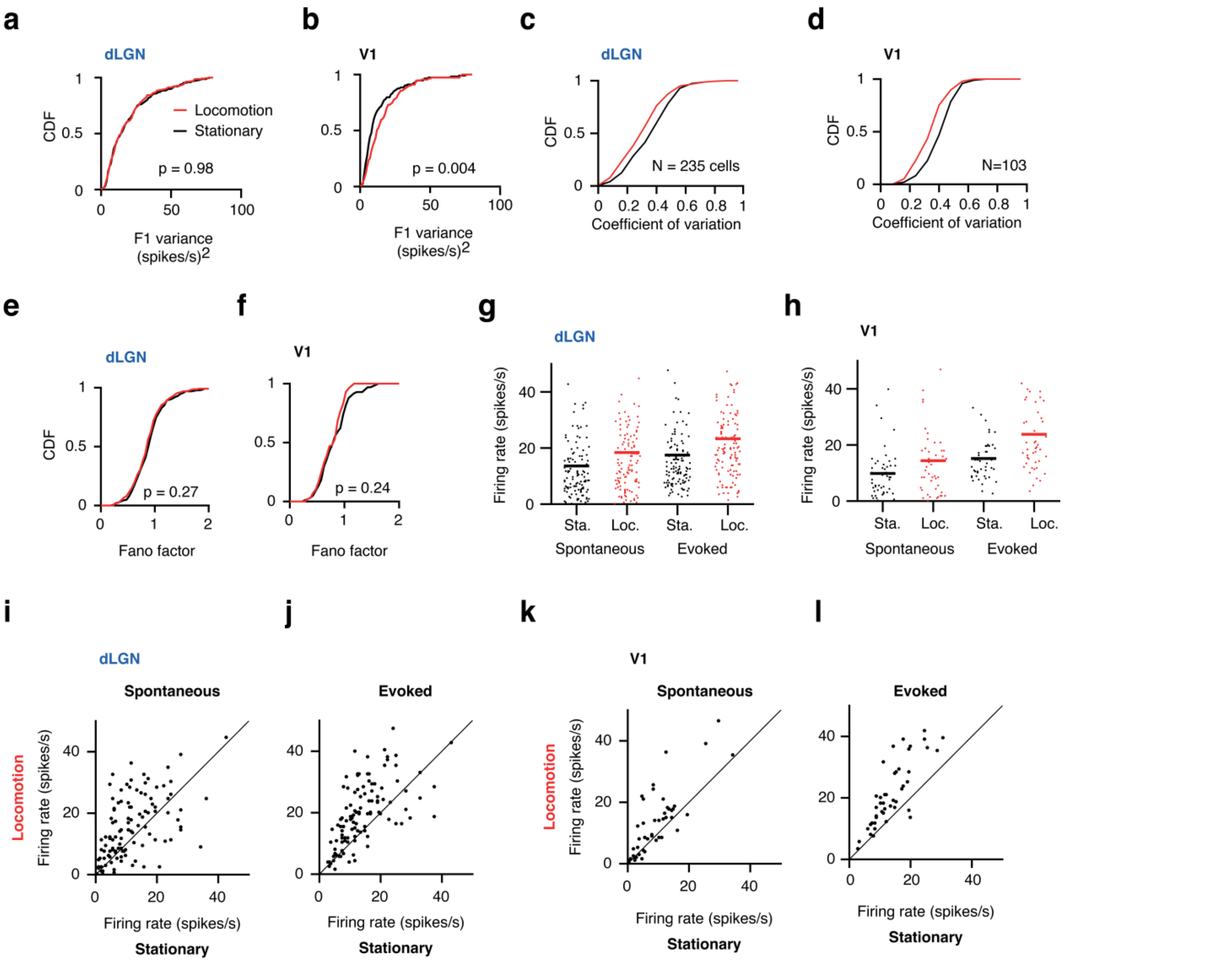

Supplementary Figure 4: (a) Mean of the F1 variance for dLGN cells across all stimulus conditions shown as cumulative distributions for stationary (black) and locomotion trials (red). **(b)** Same as a for V1 cells. **(c)** Mean F1 coefficient of variation (standard deviation/mean) for dLGN cells across all stimulus conditions that have at least 4 locomotion trials shown as cumulative distributions. **(d)** Same as c for V1 cells **(e)** Fano factor measurement between locomotion and stationary trials shown as cumulative distributions for dLGN cells. **(f)** Same as e for V1 cells. **(g)** Spontaneous and visually evoked activity (from spatial frequency experiments) of dLGN cells during stationary (black) and locomotion trials (red). **(h)** Same as g for V1 cells. **(i)** Comparison of spontaneous firing rates during stationary and locomotion conditions for dLGN cells. **(j)** Same cells in i for evoked firing rates. **(k)** Comparison of spontaneous firing rates during stationary and locomotion conditions for V1 cells. **(l)** Same cells in k for evoked firing rates.

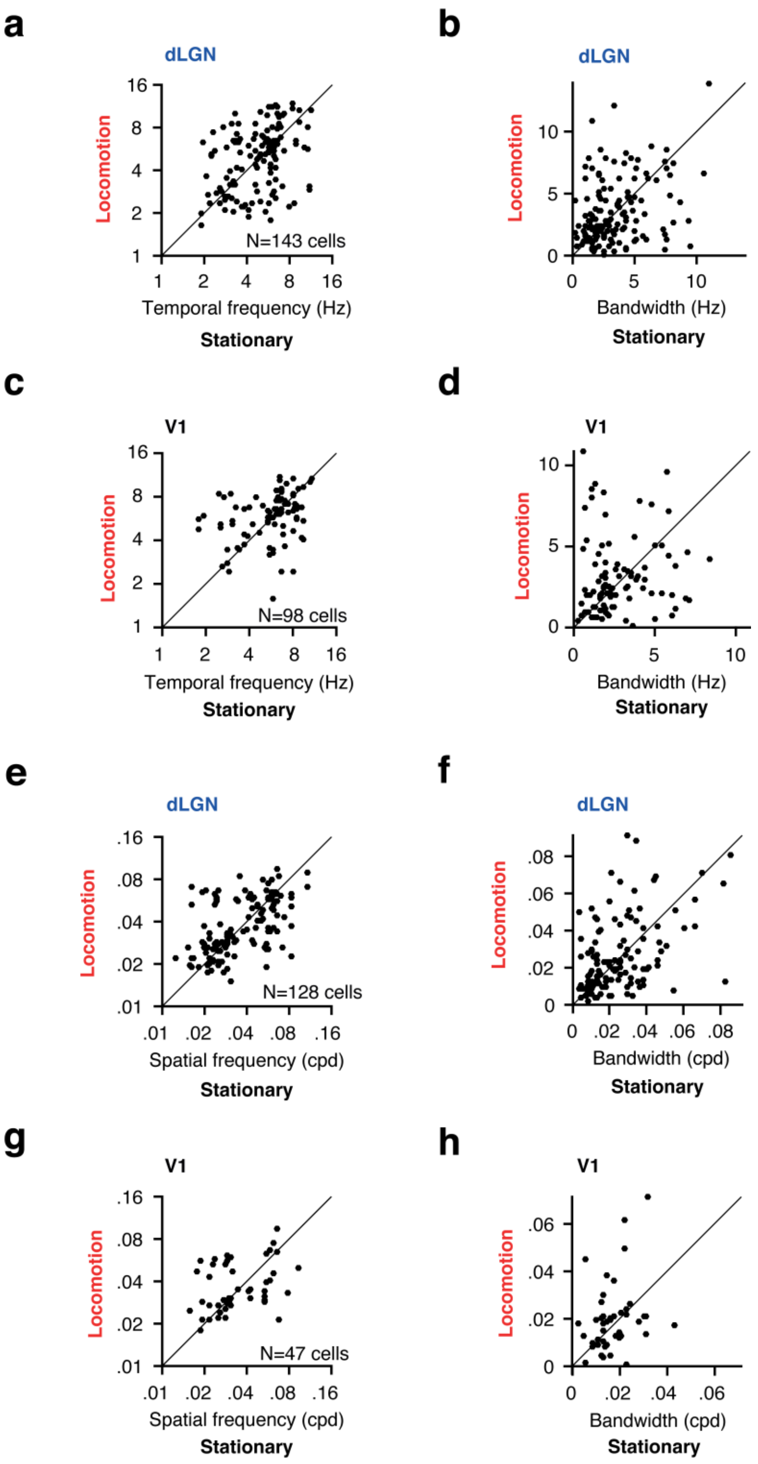

Supplementary Figure 5: (a) Preferred temporal frequency of dLGN (N=143) cells during locomotion and stationary conditions. **(b)** Tuning curve bandwidths for the same neurons in a. **(c)** Preferred temporal frequency of V1 (N=98) cells. **(d)** Tuning curve bandwidths of V1 cells in c. **(e)** Preferred spatial frequency of dLGN (N=128) cells during locomotion and stationary conditions. **(f)** Tuning curve bandwidths for the same neurons in e. **(g)** Preferred spatial frequency of V1 (N=47) cells during locomotion and stationary conditions. **(h)** Tuning curve bandwidth for the cells in g.

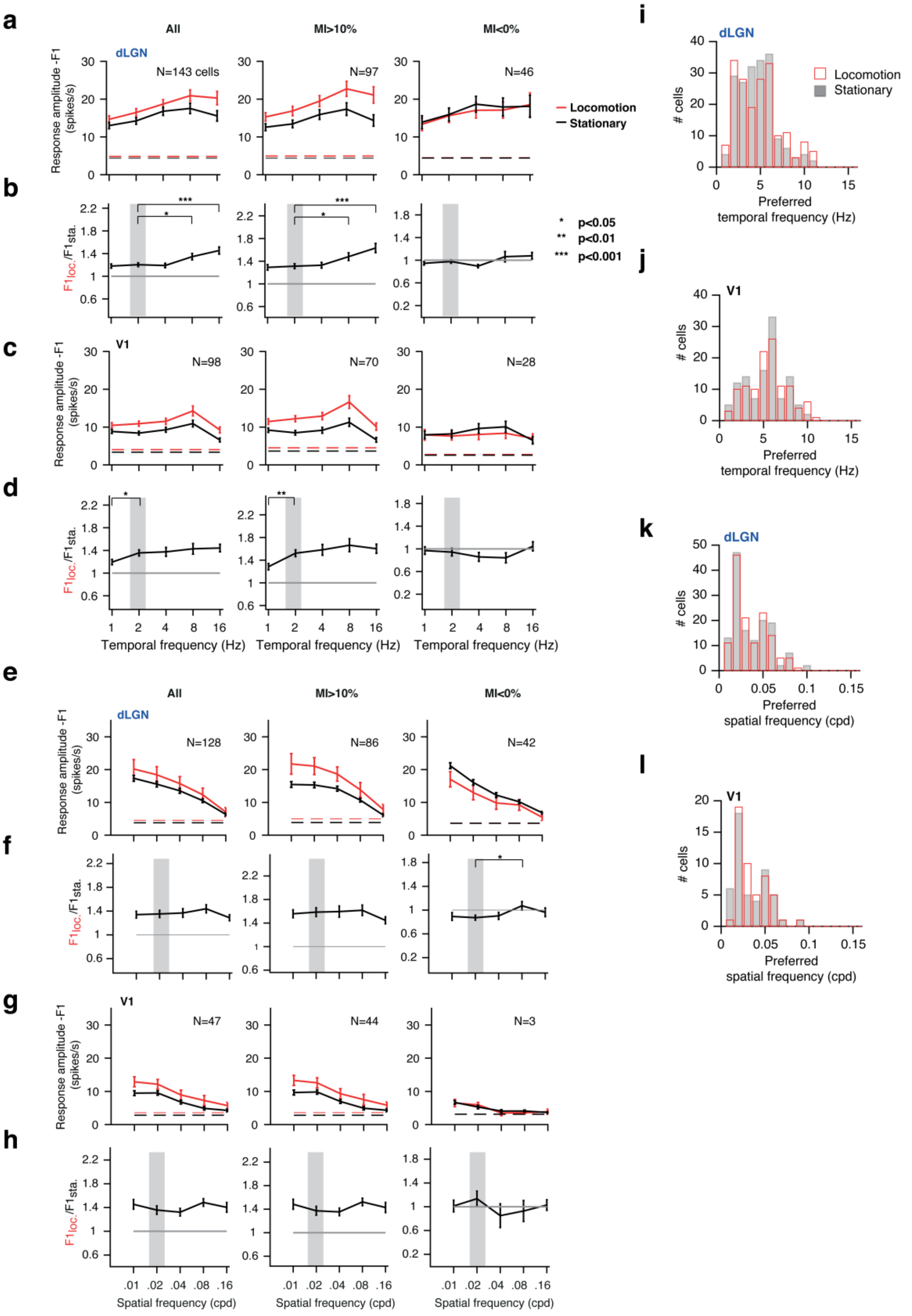

Supplementary Figure 6: (a) Population mean of the response amplitude (F1) for dLGN cells for the temporal frequency stimulus. (all cells; N=143, positively modulated cells, MI>10; N=97: negatively modulated cells MI<0; N=46). **(b)** Ratio of population mean computed over the locomotion and stationary conditions for the cells shown in a. **(c)** Same as a for V1 cells. (all cells; N=98: positively modulated cells; N=70: negatively modulated cells; N=28). **(d)** Same as b for V1 cells. **(e)** Population mean based on response amplitude (F1) for dLGN cells to spatial frequency stimulus (all cells; N=128, positively modulated cells, MI>10; N=86: negatively modulated cells, MI<0; N=42). **(f)** Ratio of population mean computed over the locomotion and stationary conditions shown for the cells shown in e. **(g)** Same as e for V1 cells (all cells; V1; N=47: positively modulated cells; N=44: negatively modulated cells; N=3). **(h)** Same as f for V1 cells. **(i)** Distribution of preferred temporal frequency of dLGN cells. **(j)** Same as i for V1 cells. **(k)** Distribution of preferred spatial frequency of dLGN cells. **(l)** Same as k for V1 cells.

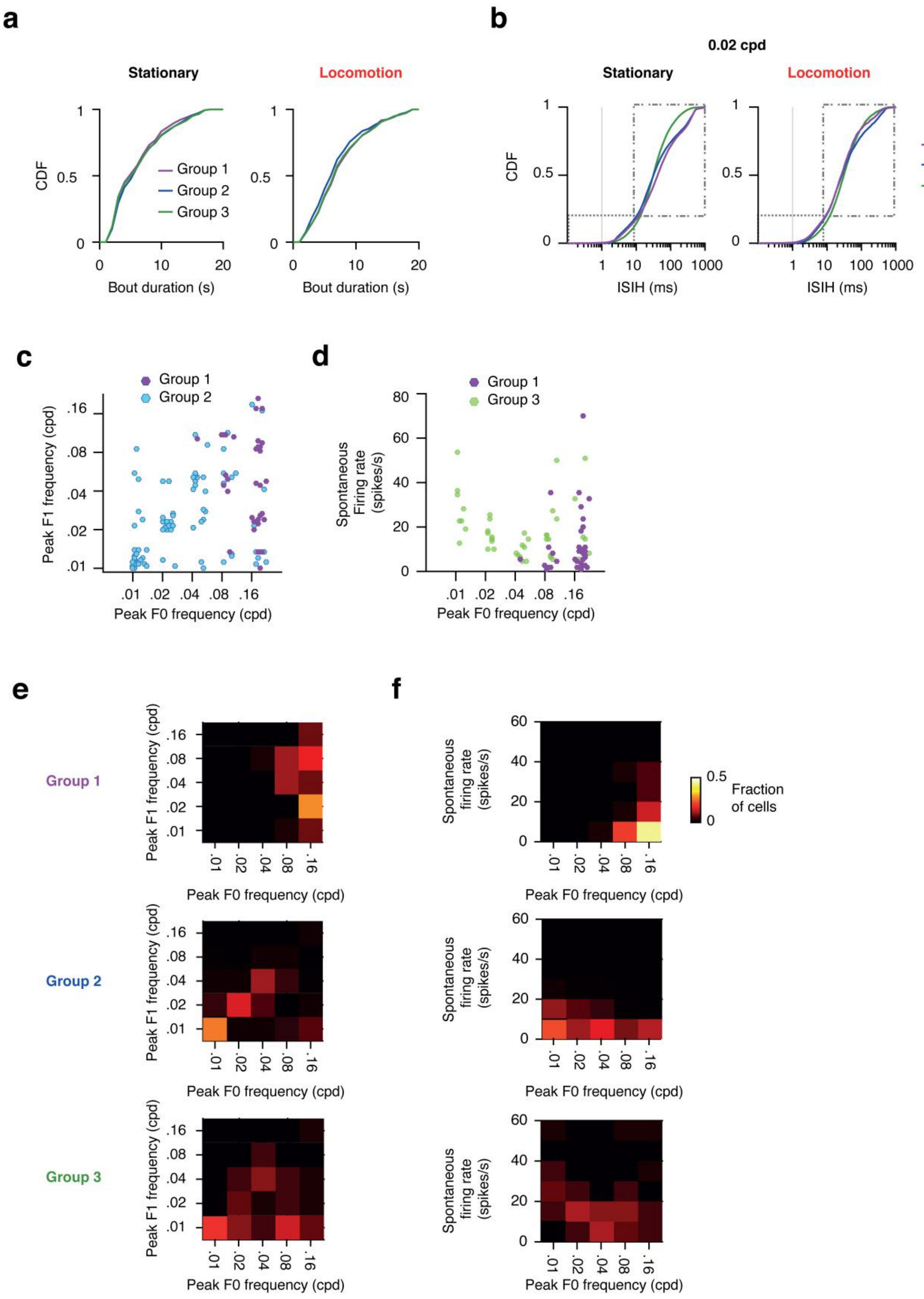

Supplementary Figure 7.(a) Cumulative distributions of stationary (left) and locomotion (right) bouts for 3 groups. **(b)** Mean inter-spike interval histograms (ISIH) for the 3 groups. High firing rate (top inlet, thin dashed, 1-10ms) and low firing rate (bottom inlet, thick dashed 10-1000ms) ISIH distributions for stationary (left) and locomotion (right) conditions with respect to the 0.02 cycle-per-degree stimulus as an example. **(c)** Comparison of peak F0 frequency with peak F1 frequency for Group 1 and Group 2. **(d)** Comparison of peak F0 frequency with baseline firing rate for Group 1 and Group 3. **(e)** Bivariate cumulative distributions as a comparison of peak F0 frequency with peak F1 frequency for the 3 groups. **(f)** Same as g as a comparison of peak F0 frequency with baseline firing rate for the 3 groups.

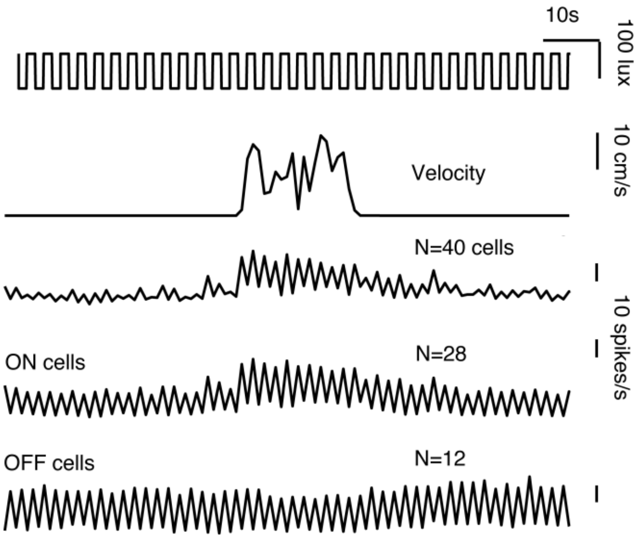

Supplementary Figure 8. Example recording session from dLGN in response to full-field contrast reversal stimuli alternating at 1 Hz (scale bar 100 lux). Stimuli luminance (top), animal velocity (2nd row, scale bar 10 cm/s), average response of visually responsive cell population (3rd row; n=40 cells, scale bar 10 spikes/s), average response of ON cells (4th row; n=28 cells, scale bar 10 spikes/s), average response of OFF cells (bottom; n=12 cells, scale bar 10 spikes/s) during locomotion and stationary conditions. Differential modulations by ON and OFF cell subpopulations.
